# Reduction of the long-term use of proton pump inhibitors by a patient-oriented electronic decision support tool (arriba-PPI): study protocol for a randomized controlled trial

**DOI:** 10.1186/s13063-019-3728-2

**Published:** 2019-11-21

**Authors:** Anja Rieckert, Annette Becker, Norbert Donner-Banzhof, Annika Viniol, Bettina Bücker, Stefan Wilm, Andreas Sönnichsen, Anne Barzel

**Affiliations:** 1 Institute of General Practice and Family Medicine, Faculty of Health, Witten/Herdecke University, Alfred-Herrhausen-Str. 50, 58448 Witten, Germany; 2 Department of General Medicine, Preventive and Rehabilitation Medicine, University of Marburg, Karl-von-Frisch Str. 4, 35043 Marburg, Germany; 3 Institute of General Practice, Centre for Health and Society, Medical Faculty, Heinrich-Heine-University Düsseldorf, Moorenstr. 5, 40225 Düsseldorf, Germany; 4 Department of General Practice and Family Medicine, Center for Public Health, Medical University of Vienna, Kinderspitalgasse 15/I, 1090 Vienna, Austria

**Keywords:** General practitioner, Evidence-based medicine, Computerized clinical decision support system, Deprescribing, Proton pump inhibitors

## Abstract

**Background:**

Proton pump inhibitors (PPIs) are increasingly being prescribed, although long-term use is associated with multiple side effects. Therefore, an electronic decision support tool with the aim of reducing the long-term use of PPIs in a shared decision-making process between general practitioners (GPs) and their patients has been developed. The developed tool is a module that can be added to the so-called arriba decision support tool, which is already used by GPs in Germany in routine care. In this large-scale cluster-randomized controlled trial we evaluate the effectiveness of this arriba-PPI tool.

**Methods:**

The arriba-PPI tool is an electronic decision support system that supports shared decision-making and evidence-based decisions around the long-term use of PPIs at the point of care. The tool will be evaluated in a cluster-randomized controlled trial involving 210 GP practices and 3150 patients in Germany. GP practices will be asked to recruit 20 patients aged ≥ 18 years regularly taking PPIs for ≥ 6 months. After completion of patient recruitment, each GP practice with enrolled patients will be cluster-randomized. Intervention GP practices will get access to the software arriba-PPI, whereas control GPs will treat their patients as usual. After an observation period of six months, GP practices will be compared regarding the reduction of cumulated defined daily doses of PPI prescriptions per patient.

**Discussion:**

Our principal hypothesis is that the application of the arriba-PPI tool can reduce PPI prescribing in primary care by at least 15% compared to conventional strategies used by GPs. A positive result implies the implementation of the arriba-PPI tool in routine care.

**Trial registration:**

German Clinical Trials Register, DRKS00016364. Registered on 31 January 2019.

## Background

Prescriptions of proton pump inhibitors (PPIs) have been increasing considerably in recent years in many countries. According to the German drug prescription report, a total of 3.7 billion defined daily doses (DDD) of PPIs were prescribed in Germany in 2015. Thus, PPIs are one of the most commonly prescribed drugs [1]. Even though the number of PPI prescriptions slightly decreased from 2016 to 2017, the number of prescribed PPIs still remains high. The halt of the rising trend might be due to the recent discussion around possible side effects caused by PPIs when used for long periods [2].

Positive evidence exists regarding the effectiveness of PPIs in the treatment of gastrointestinal ulcers [3], eradication therapy [4], reflux disease [5], and gastric pre-malignant lesions [6]. However, PPIs are also increasingly used as a means of protecting the stomach in polypharmacy patients (i.e. the current intake of several drugs [7]) and in combination with non-steroidal antirheumatic drugs or platelet aggregation inhibitors. Furthermore, they are used in patients suffering from nun-ulcer dyspepsia and for stress ulcer prophylaxis during hospital stay [1, 8]. PPIs should be used for short periods and only few indications justify their long-term use. Even though long-term use without indication is considered inappropriate [9], PPIs are frequently overused as lifestyle drugs [10]. Long-term use of PPIs poses potential risks [9], such as interactions with other drugs or side-effects [11–13]. Once prescribed, withdrawing PPIs seems to be difficult due to a potential rebound effect reactivating dyspeptic complaints [14]. Apart from the potential risks, the frequent use of PPIs contributes to substantial costs for the healthcare system [1].

Given the frequent use and overuse of PPIs, withdrawing PPIs is important and supporting strategies for GPs are needed [15]. Deprescribing is “the process of withdrawal of an inappropriate medication, supervised by a health care professional [ …]” [16]. A recent Cochrane review identified the benefits and harms of deprescribing for chronic PPI use. Six studies were included; five of them deprescribed PPIs on-demand, whereas one abruptly discontinued PPIs. Overall, a significant reduction in the number of PPIs taken could be achieved. The deprescribing of PPIs led to side effects such as significantly more gastrointestinal complaints [17]. However, a recently developed guideline to support deprescribing of PPIs concluded that PPIs can be withdrawn without causing any major clinical harm [15]. Still, there are not enough data on the long-term benefits or harms of PPI withdrawal and the cost/resource use of the interventions is not known. Furthermore, the patient was not involved in the deprescribing process [17].

Involving the patient into the deprescribing process is important and it has been shown that deprescribing interventions are most effective when they involve the patient [18]. Furthermore, decision aids can support patients in their treatment decisions, enhance informed, value-based choices, and improve patient–practitioner communication compared to usual care [19].

The electronic decision support tool arriba is widely used in Germany and has been originally developed to support shared decision-making (SDM) processes in GP practices in the prevention of cardiovascular diseases. Arriba was developed by Institutes of General Practice of several German universities and is nowadays managed by the independent and non-profit arriba cooperative society. It is based on the principles of evidence-based and individual patient-oriented medicine [20]. Today, arriba consists of several modules that have been scientifically evaluated previously [20–25].

Given the long-term overuse of PPIs and their potential risks, there is a strong need for effective interventions optimizing the long-term use of PPIs. Therefore, an additional module for the arriba tool has been developed. The arriba-PPI tool is targeted at the primary care setting to support GPs to identify and reduce inappropriate long-term prescribing of PPIs in a SDM process with their patients. It presents options and their evidence base in an easy to understand way and offers practical behavioral advice and individualized messages for patients. In line with the MRC framework for complex interventions [26], we evaluated the arriba-PPI tool in a feasibility study before the start of this trial [27].

## Methods/Design

We followed the Standard Protocol Items: Recommendations for Interventional trials (SPIRIT checklist) in designing the study protocol (see Additional file 1). As recommended by the MRC framework [26], our project incorporates three of the four elements: development; piloting; and evaluation. Upon positive evaluation, the arriba-PPI tool will be implemented (fourth element of the MRC framework). This study protocol mainly focuses on the evaluation.

### Objectives of the study

Our principal hypothesis is that a patient-oriented strategy of medication reduction using the arriba-PPI tool in primary care practices reduces PPI prescriptions by at least 15% in comparison to conventional consultations over a period of six months (see Table 1).

**Table 1:** PICO research question of the arriba-PPI trial

PICO-Item	Research question
P (patient)	Patients aged ≥ 18 years regularly using PPIs over ≥ 6 months in primary care
I (intervention)	GPs of enrolled patients with access to the arriba-PPI tool
C (comparison)	GPs of enrolled patients without access to the arriba-PPI tool providing care as usual
O (outcome)	Cumulated defined daily doses of PPI per study patient after 6 months

Our secondary objective is to evaluate the effectiveness of the implementation of the arriba-PPI tool in our extension study (6–12 months).

Furthermore, we aim to describe the GPs’ and the patients’ experiences in using the arriba-PPI tool within a primary care consultation in two sub-studies.

### Trial design and setting

**Fig. 1:**
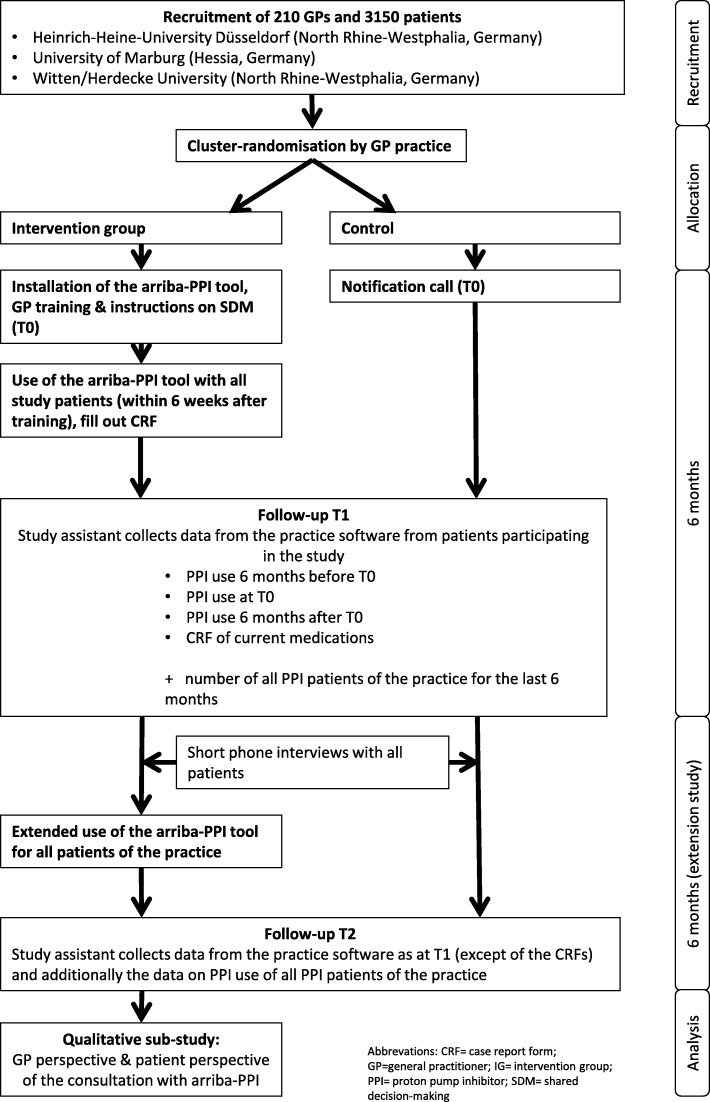
Course of the study

The arriba-PPI trial is a national multicenter cluster-randomized controlled trial with an observation period of one year to reduce long-term prescription of PPIs. It will be conducted in Germany in the mid- and north Hessia and Westphalia-Lippe regions. Three study centers will be involved: the Institute of General Practice of Marburg University; Institute of General Practice of Heinrich-Heine-University Düsseldorf; and Institute of General Practice and Family Medicine of Witten/Herdecke University. The arriba-PPI trial will be located in the primary care setting. Details on the study procedure are outlined in Fig. 1.

### Ethics approval

The study has been approved by the three local ethics committees (see “Declarations”).

### Recruitment of GP practices and patients

Before the recruitment of GP practices and patients, recruitment-regions will be selected. In these prespecified regions, all GPs will be informed about the study and will be invited to participate. All GP practices will obtain a written invitation (letter and/or fax) and will be followed up by phone calls. The study design will be presented at several network meetings for GPs, where the GPs will be asked to participate. Additionally, the health insurance BARMER will identify GP practices with a higher prescription rate of PPIs compared to the median prescription rate of all practices of the BAMER database. BARMER will contact these practices twice via mail and invite them to participate.

Participating GP practices will be visited by a research assistant from the study center providing detailed information about the study before patient recruitment. GP practices will then invite all consecutive patients with a PPI prescription consulting the practice to participate in the study and will inform them about the study orally and in writing. After completing recruitment, the practice will be randomized.

### Inclusion and exclusion criteria

GP practices will need to meet the following inclusion criteria to participate: German as the predominant language in patient communication; the ability to collect prescription data in an electronic health record (EHR) as a technical prerequisite; and the willingness to provide PPI prescription data collected in the EHR. Practices will be excluded if they only treat narrowly defined patient groups or provide services only (e.g. alternative or complementary treatments), do not regularly prescribe PPIs, or do not use an EHR.

Patients with a regular prescription of PPIs of ≥ 6 months will be included. We defined regular prescription as taking at least one PPI pill daily or as taking regularly several PPI pills per week (such as four pills per week/every other day). Furthermore, patients will have to be aged ≥ 18 years and give informed consent according to the declaration of Helsinki. Patients will be excluded from the study if they do not want to participate, cannot provide informed consent, or are not able to communicate in German. Only patients who have access to the practice will be included, since the arriba-PPI tool is only available for computers. Lastly, patients are excluded if their PPIs are prescribed for limited time periods or only as required.

### Randomization

The GP practice is regarded as the unit of randomization. Each participating GP practice with the recruited patients will be randomized to either having access to the arriba-PPI tool (intervention) or not and performing care as usual (control). We decided to apply cluster randomization in order to avoid contamination if the GP would use the tool for some patients but not for others as we expect that a learning effect will take place. After completion of patient recruitment, the GP practice will notify the corresponding study center providing the number of patients recruited. The practice will then be randomized using computerized sequence generation with a simple randomization scheme generated by the random package of the program R [28]. Randomization will be stratified by study center. Randomization lists will be kept closed. To assure concealment of allocation, no patients can be included once recruitment is completed and randomization has been performed.

### Blinding

Due to the nature of the intervention, neither GPs nor patients can be blinded. For practical reasons, study personnel cannot be blinded either. However, all analyses will be conducted by a blinded statistician.

### Intervention

The intervention consists of the arriba-PPI tool applied during a regular or extra patient contact in the GP practice. Before the intervention, study personnel will visit the intervention group practices to provide training for GPs and practice nurses that comprises the use of PPIs in general, the SDM processes, the withdrawal of drugs, and how to use the arriba-PPI tool. GPs are supposed to use the arriba-PPI tool with their participating study patients for the following six months and, subsequently, the arriba-PPI tool application shall be used for all patients with a PPI prescription for another six months.

The application of the arriba-PPI tool requires the installation of the arriba software on one or several computers of the GP practice to enter relevant patient data including name, gender, PPI substance, dose, and indication. Once patient data are entered, a choice of four sections is available represented by the following buttons: traffic light; weighing scale; procedure; information; and print.

The decision aid is displayed as a traffic light system to clarify whether stopping PPIs is recommended or not. Green indicates the clear recommendation for a withdrawal, yellow indicates that withdrawal usually is recommended, and red indicates that withdrawal usually is not recommended. The weighing scale provides arguments for and against withdrawal (such as long-term harm, short-term complaints, social constraints). GPs are supposed to discuss with their patient the pros and cons for taking PPIs, taking the patient’s preferences into consideration. Depending on the decision made by the patient and GP, the software provides suggestions for next steps to take, in particular the measures to be taken when complaints arise during withdrawal. Finally, the patient will get an individualized printout covering information on long-term effects of the drug, a withdrawal plan with dosing steps, follow-up appointments, and so on.

### Control

GP practices participating in the control group of the study will provide care as usual for 12 months. GP practices will not make any extra appointments with their patients for this study.

### Measurements

The primary endpoint is the cumulated DDDs of PPIs per study patient at six months (T1). Secondary endpoints are the proportion of PPI patients in the practice during the six months after allocation (T1), the cumulated DDDs of PPIs per study patient during the 12 months after allocation (T2), the proportion of PPI patients in a practice during the time span of 6–12 months after allocation (T2), and the average accumulated total DDDs of PPIs in a practice for all patients during the time span of 6–12 months after allocation (T2).

**Fig. 2:**
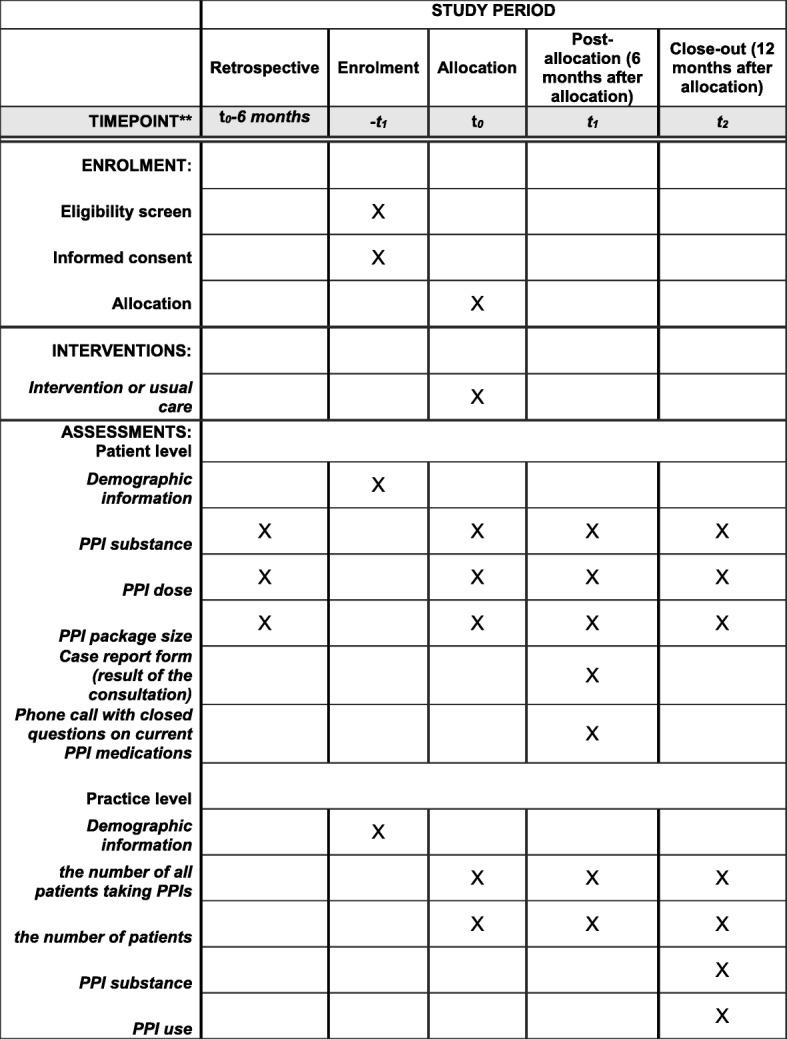
Schedule of enrolment, interventions, and assessments for the arriba-PPI trial according to SPIRIT

For outcome measurement, information on PPI prescription (substance, dose, package size) per patient will be recorded from the practice software for a time span of six months before T0 to 12-month follow-up. Furthermore, the number of all patients taking PPIs per practice and the number of patients per practice will be assessed for the time span of T0 to 12 months after allocation (Fig. 2).

To evaluate the real utilization of the arriba-PPI tool and to control for confounding, GPs from the intervention group will fill out a case report form for each patient after consultation with the arriba-PPI tool, which provides information on the original indication, the result of the consultation, and on medication changes. At T1, GPs will be asked whether there has been a change in the PPI medication for each individual study patient and, if so, why. Additionally, after T1 study centers will call all patients for a short-structured phone interview based on mainly closed questions to gain information about the current PPI medications and other medications for gastric problems they are taking to monitor medication shifts, e.g. into self-medication (over-the-counter medication). In the intervention group, patients will also be asked whether the arriba-PPI tool was used during consultations.

Finally, demographic data from all participating GPs will be collected before randomization using a written questionnaire.

### Monitoring

Personnel not involved in this study will perform monitoring according to a pre-specified manual. In short, the monitors will control whether informed consent forms from all participating patients and practices are correctly filled out and signed. For a randomly chosen 15% sample, the SDM process with the application of the arriba-PPI tool will be monitored. At six months (T1), we will control in 15% of all patients whether they fulfilled the inclusion criteria before randomization. Furthermore, 15% of all data entered into SPSS will be checked by a second person not involved in the study.

### Sample size calculation

We based our sample size calculation on our primary endpoint. According to data of the statutory health insurance AOK Hessen, the average DDD of all PPI prescriptions per GP practice (averaged across all GP practices) accounts to 8244.47 per quarter with a standard deviation of 7850.89 (variation coefficient 0.95). This value is based on an average number of patients with PPI prescriptions of 56.20 per GP practice. Therefore, an average of DDD of PPIs of 146.7 per patient is assumed (8244.47/56.2). Taking into account a variation coefficient of 0.95, the standard deviation is 139.7 DDD of PPIs on a patient level. We consider a reduction by 15% as relevant, which would correspond to a difference of 22 DDD (15% of 146.7) between the control and intervention groups at T1. According to the sample size calculator for cluster-randomized trials of the University of Aberdeen’s Health Services Research Unit [29], we will need 204 GP practices with 15 patients each if we want to prove such a difference with an intraclass correlation coefficient of 0.1 [30], a significance level of 0.05, and a power of 80%. The primary outcome relates to patient-level data. Thus, we adapted our calculation to the cluster structure (patients in GP practices). The number of practices refers to the number that needs to be randomized. To cover for drop-out practices after randomization, we increased the number of practices to be recruited to 210. In addition, patients might leave the study at their own request after inclusion in the study but before receiving the actual intervention. We decided to have a buffer considering non-predictable dropouts. Therefore, each study practice is required to recruit at least 15 patients but not more than 25 patients, as differences in cluster size can have a negative influence on intracluster variability. Practices will include patients in the study according to the chronological order in which the patients signaled their willingness to participate in the study. By setting this range of patients, the probability that the number of patients per practice varies only minimally increases. If 15 potential study patients cannot be enrolled in the study within the timeframe for recruitment, the study practice and its recruited study patients will still be included in the analysis.

### Data management and statistical analysis

Data will be recorded into SPSS at each study center and transferred to the blinded trial statistician. We will use intention-to-treat and per-protocol analyses. The primary outcome will be analyzed by multilevel analyses with the statistical program R following intention-to-treat principles [31]. These analyses take into account the clustering of patients by practices and allow for different modelling of predictors, e.g. group affiliation as fixed and/or random effect. Furthermore, these analyses allow adjustment for variables which showed differences between the intervention and control groups despite randomization and which are considered to be of prognostic importance. In case of missing variables, suitable imputation methods will be used [32]. We will perform sensitivity analyses (worst case, best case, complete case) in order to check for the influence of missing data on the results.

The evaluation for the secondary outcomes will also be done by multilevel analyses by adopting covariates on patient and/or cluster level. Elaborated evaluations in multivariate procedures allow a more detailed analysis of prescription behavior. All statistical tests will be two-tailed and an alpha of 5% will be used throughout. Besides statistical significance, effect sizes will also be evaluated [33].

### Qualitative sub-study

In order to explore the experiences of the usage of the arriba-PPI tool, the adoption of the advice, and the SDM process, we will conduct in-depth interviews with participating GPs and patients. Therefore, we will invite randomly selected GPs and patients to separate focus group meetings or individual interviews. Interviews will be conducted by means of a semi-structured interview guideline, recorded, anonymized, and transcribed verbatim. The qualitative text will be analyzed by thematic qualitative text analysis in multidisciplinary groups.

## Discussion

In this study, we conduct a large multicenter randomized controlled trial to analyze the effectiveness and the implementation of the arriba-PPI intervention with the aim to reduce the long-term use of PPIs applying a patient-oriented SDM process. Because of the pragmatic trial design, we cannot assure blinding of staff and participants. However, the trial statistician remains blinded during analysis of data.

Even though withdrawal of inappropriate drugs is recognized as being important, it remains challenging in the practice. Studies show that there are a variety of patient-related [34] and prescriber-related [35] factors hindering deprescribing in general. Especially in polypharmacy patients, changes in medication can impact on other drugs that the patient is currently taking [36]. These barriers might also be encountered in our trial. Therefore, in addition to the effectiveness of the arriba-PPI tool, we will explore both, the patients’ perspective and the GPs’ perspective on deprescribing PPIs with the arriba-PPI tool. These qualitative evaluations will provide a better understanding of the effects of the implementation and shed light on optimizations needed for future implementation.

In this trial we will include GPs who represent the target population working in usual German GP practices. However, GPs that consent to participate in this trial possibly have a higher affinity towards the use of an electronic decision support tool than GPs not participating in our study. Furthermore, they might have a different attitude towards PPI use and be more motivated to deprescribing PPIs. Patients in this trial in general represent patients that GPs encounter in everyday practice. However, patients that consent to participate in the study might be more motivated to make changes compared to their peers. The trial takes place in Germany, limiting the generalizability of the results to other healthcare settings. Furthermore, we did not plan any full health economic evaluation. Despite these limitations, this study will provide valuable insights into the effectiveness of deprescribing PPIs supported by the arriba-PPI tool and its impact on clinical practice.

This study addresses the inappropriate use of PPIs, a drug class that is widely overused in many countries [1]. We expect the recently developed electronic decision support tool arriba-PPI to encourage GPs to broach the issue with their patients. The fact that the arriba-PPI tool is part of the arriba software package will make implementation easier.

## Trial status

This manuscript presents the version of 26 June 2019 of the arriba-PPI protocol. Recruitment started in December 2018 and is expected to be completed by 15 July 2019.

## Supplementary information

**Additional file 1.** SPIRIT Checklist.

**Additional file 2.** Patient consent form in German/not translated.

## Data Availability

Not applicable.

## Abbreviations

DDD: Defined daily dose
EHR: Electronic health record
GP: General practitioner
PPI: Proton pump inhibitor
SDM: Shared decision-making
