# Supplementary material for: Reduction of the long-term use of proton pump inhibitors by a patient-oriented electronic decision support tool (arriba-PPI): study protocol for a randomized controlled trial
Source: Trials. 2019 Nov 21;20:636. doi: 10.1186/s13063-019-3728-2 (PMC6868794; doi:10.1186/s13063-019-3728-2)
Supplement: Supplementary file 2 — Additional file 2. Patient consent form in German/not translated. [file 13063_2019_3728_MOESM2_ESM.docx]

| **Zurück ins Studienzentrum** |  |  |
| --- | --- | --- |

| **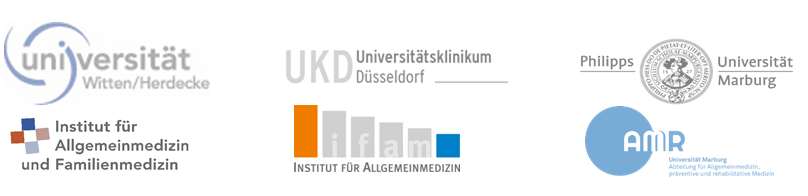** |
| --- |
| **Patienten-Einverständniserklärung zur Studienteilnahme** |

**„arriba®-med:** **Evaluation einer versorgungsnahen Intervention zur Optimierung des Einsatzes von PPI “**

Ich, Frau/Herr ______________________________ (**Namen bitte deutlich schreiben!**)

wurde heute vollständig über Wesen und Bedeutung der oben genannten wissenschaftlichen Studie aufgeklärt. Ich habe die Studieninformation sowie die nachfolgend abgedruckten Rechte über meinen Datenschutz gelesen und verstanden. Ich hatte ausreichend Möglichkeit, Fragen zu stellen, und habe die Antworten verstanden.

Ich weiß, dass die Teilnahme freiwillig ist. Meine Zustimmung kann ich jederzeit ohne Angabe von Gründen widerrufen, ohne dass sich dieser Entschluss nachteilig auswirken wird.

Mir ist bekannt, dass bei dieser Studie personenbezogene Daten über mich erhoben, pseudonymisiert gespeichert und quantitativ ausgewertet werden. Die personen- und praxisbezogenen Daten werden nach Studienende gelöscht. Die pseudonymisierten, ausgewerteten Daten werden nach Beendigung der Studie 10 Jahre im Studienzentrum gespeichert.

Ich erkläre mich hiermit einverstanden, an der Studie „arriba®-med“ teilzunehmen und gestatte die Weitergabe meiner Kontaktdaten an das Studienzentrum für eine telefonische Kontaktaufnahme durch einen Studienmitarbeiter.

Ich bin außerdem damit einverstanden, dass personenbezogene Gesundheitsdaten von dem Arzt für diese Studie über mich erhoben werden, soweit dies für die ordnungsgemäße Durchführung der Studie notwendig ist. Insoweit entbinde ich diesen Arzt von der Schweigepflicht. (Falls nicht gewünscht, bitte streichen.)

Eine Kopie des Informationsblattes und dieser Einverständniserklärung habe ich erhalten.

**Ort, Datum Unterschrift Patient/Patientin**

Ich bestätige, dass ich oben genannten Patienten, der sein Einverständnis für die Teilnahme gegeben hat, bezüglich Zweck, Nutzen und Art der Studie aufgeklärt habe.

**Ort, Datum Unterschrift der aufklärenden Person**
